# Personality Effects on Chinese Public Preference for the COVID-19 Vaccination: Discrete Choice Experiment and Latent Profile Analysis Study

**DOI:** 10.3390/ijerph19084842

**Published:** 2022-04-15

**Authors:** Jinzi Zhang, Pu Ge, Xialei Li, Mei Yin, Yujia Wang, Waikit Ming, Jinhui Li, Pei Li, Xinying Sun, Yibo Wu

**Affiliations:** 1 School of Humanities and Social Sciences, Harbin Medical University, Harbin 150081, China; 15045118988@163.com (J.Z.); dryinmei@163.com (M.Y.); 18145681982@163.com (Y.W.); 2 Institute of Chinese Medical Sciences, University of Macau, Macau 999078, China; 17853140673@163.com; 3 School of Pharmaceutical Sciences, Shandong University, Jinan 250012, China; lixialei0929@163.com; 4 Department of Infectious Diseases and Public Health, Jockey Club College of Veterinary Medicine and Life Sciences, City University of Hong Kong, Hong Kong 999077, China; wkming2@cityu.edu.hk; 5 School of Journalism and Communication, Jinan University, Guangzhou 510632, China; lijinhui@jnu.edu.cn; 6 Hong Kong Institute of Health Education, Hong Kong 999077, China; 18310590509@163.com; 7 School of Public Health, Peking University, Beijing 100191, China

**Keywords:** discrete choice experiment, COVID-19, vaccine preference, latent profile analysis, big five personality traits

## Abstract

**Objective:** This study aims to investigate the differences in public vaccination preference for the COVID-19 vaccine with different personality characteristics. **Methods:** Based on the Big Five Personality Inventory (BFI-10), a total of 1200 respondents were categorized by personality characteristics using Latent Profile Analysis (LPA). The preference of members the public with different personality characteristics for COVID-19 vaccination was investigated based on a discrete choice experiment (DCE). **Results:** All respondents were divided into three groups, named the General and Stable type (79.67%), Conscientious and Agreeable type (9.5%), and Open and Extroverted type (10.83%). For the percentage importance of vaccine attributes, both the General and Stable type and Conscientious and Agreeable type respondents considered cost to be the most important (41.93% and 34.95% respectively). However, the Open and Extroverted type respondents considered efficacy as the most important (31.05%). In our conditional logit model (CLOGIT), for vaccine adverse effects, the General and Stable type and Conscientious and Agreeable type respondents preferred “very mild”, while the Open and Extroverted type preferred “mild” (OR:1.108, 95%CI 0.977–1.256). The Open and Extroverted type had a higher willingness to pay (WTP) for the most preferred vaccine level compared to the other types. **Conclusions:** The Open and Extroverted respondents have the highest willingness to vaccinate. The General and Stable type and Conscientious and Agreeable respondents think that the cost of the vaccine is the most important attribute, and prefer the mildest side effects. The Open and Extroverted type think that vaccine efficacy is the most important attribute, prefer “mild” side effects, and have higher willingness to pay for their favorite vaccine level.

## 1. Introduction

By the end of 18 March 2022, COVID-19 had infected more than 464 million people across 221 countries and territories, with more than 6.06 million deaths worldwide [1]. It is difficult to completely isolate the source of infection because of the rapid spread of COVID-19 and the large number of susceptible people. Therefore, massive vaccination coverage is considered as a prerequisite to achieving herd immunity and therefore curbing the COVID-19 pandemic [2]. However, the biggest challenge facing the realization of group immunity is ensuring a sufficiently high vaccination rate. Some studies have estimated the vaccination rate required for the realization of group immunity to be between 67% and 95% [3,4,5]. The COVID-19 vaccines differ from previous vaccines in many respects: development speed, innovativeness of the techniques used, uncertainty regarding the magnitude and extent of their effectiveness, and potential side effects [6,7,8]. Therefore, public preference for COVID-19 vaccination needs to be studied to achieve population immunity to the SARS-CoV-2 virus. By September 2021, many different types of COVID-19 vaccines had been developed [6,8], and vaccination rollouts had started in a few countries.

China is one of the few countries that can guarantee the planned immunization with its abilities. Domestic vaccines account for more than 95% of China’s vaccinations [9]. As early as the initial stage of the COVID-19 pandemic, Academician Chen Wei’s team took the lead in carrying out a clinical trial of a COVID-19 vaccine, and then countries also carried out the research and development of COVID-19 vaccines one after another [10]. Because of the novelty of this pathogen, it is impossible to predict the safety and effectiveness of all kinds of COVID-19 vaccines [11]. In February 2020, China deployed various technical approaches in the research and development of COVID-19 vaccines, including inactivated vaccines, adenovirus vector vaccines, genetically engineered recombinant subunit vaccines, attenuated vector vaccines of the influenza virus, and nucleic acid vaccines (mRNA and DNA vaccines) [12]. China has conditionally approved the marketing of three inactivated COVID-19 vaccines developed by Sinopharm (Beijing, China), Sinopharm (Wuhan, China), and Sinovac Biotech Co., Ltd. (Beijing, China); and one adenovirus-vectored COVID-19 vaccine developed by CanSino Biologics Inc. (Tianjin, China). Among the inactivated vaccines, BBIBP-CorV developed by Sinopharm (Beijing) and CoronaVac developed by Sinovac Biotech Co., Ltd. finalized and passed the WHO PQ and supported global epidemic control [13]. By March 2022, China had provided more than 2.1 billion doses of vaccines to more than 120 countries and international organizations, accounting for one-third of total vaccine use in the world outside China [14].

However, with the reports of side effects after vaccination, especially following reports of adverse events after shots of the mRNA vaccination [15], sudden deaths secondary to shots of mRNA COVID-19 vaccines in Norway [16], and so on, the public remains skeptical about the safety and efficacy of the COVID-19 vaccines. Furthermore, there is the emergence of SARS-CoV-2 variants, such as the Alpha variant that was first identified in the United Kingdom, the Beta variant that was identified in South Africa, the Gamma variant that was identified in Brazil, the Delta variant that was verified in India, and a new SARS-CoV-2 variant of concern (VoC), Omicron, that was discovered in November 2021 [6,17]. There are also conflicting reports on whether COVID-19 vaccines have consistently retained high efficacy for each of the COVID-19 variants preceding Omicron. For example, a preprint paper released by Public Health England showed that the Pfizer and the AstraZeneca vaccines’ single dose is 33% effective against variants from India [18]. Whether the current vaccines can protect the public from the SARS-CoV-2 virus and variants is of public concern.

Personality represents a series of personality characteristics, thinking patterns, and habitual behaviors within individuals, and influences individuals’ responses to external stimuli and interactions with other people in society. It is generally believed that the Big Five model covers a wide range of personality dimensions and has advantages in studying the individual or interactive influence of personality traits on healthy behaviors [19]. The five factors of personality are Neuroticism, Extraversion, Openness, Agreeableness, and Conscientiousness. Among them, Neuroticism reflects the stability of an individual’s emotional state and the tendency of their inner experience (i.e., poor emotional stability, anxiety, depression, or impulsiveness). Extroversion reflects the strength and dynamic characteristics of a person’s nervous system (i.e., energetic, extroverted, and sociable). Openness reflects the openness, wisdom, and creativity of individuals to experience (i.e., creativity, curiosity, and willingness to accept new ideas). Agreeableness reflects humanity’s humanitarianism and interpersonal orientation (i.e., trustworthiness, altruism, and sympathy). Conscientiousness embodies the abilities of self-discipline, motivation and sense of responsibility for achievement (i.e., self-discipline, due diligence, and consideration) [20].

Studies have shown that the Big Five personality test is related to psychological and behavioral responses in the face of the COVID-19 pandemic. For example, Conscientious and Agreeable individuals have higher compliance with preventive measures in COVID-19 and are the most likely to adhere to preventive measures [21]. Extraversion was negatively correlated with the degree of adherence to the guiding principles, and people with high Extraversion experienced more difficulties in complying with measures to prevent transmission in COVID-19 [21]. People with higher Neuroticism would pay more attention to COVID-19, pay more attention for a long time, feel more likely to be unsafe, and fear the economic loss caused by the crisis [22]. Open-minded individuals were more likely to adapt to new situations under the epidemic [23]. Previous literature suggests that more attention should be paid to personality in the study of vaccination attitudes and behaviors [24]. The reason is that the exploration of the personality root of the willingness to vaccinate can help to determine methods to reduce hesitation around vaccination and its influence on public health. At the same time, the determination of personality characteristics can adjust the acceptance of vaccination, which can improve the existing theories or put forward new theories, which may ultimately help to improve our understanding of the hesitation of vaccination [24].

In summary, the purpose of this study was to explore the Chinese public’s vaccination preference for COVID-19 and the differences in this preference among people with different personality characteristics based on a discrete choice experiment (DCE) and latent profile analysis (LPA), to provide ideas for formulating more accurate vaccination strategies for COVID-19.

## 2. Materials and Methods

### 2.1. Study Design

We designed 11 different scenarios, each of which included multiple options of randomly combined attributes and levels. Questionnaires were created by Sawtooth software’s Lighthouse Studio modules (version9.12.0, Sawtooth software, Orlando, FL, USA) for general interviews and choice-based conjoint (CBC) scenario design. Respondents were aged 18 to 85 years. We aimed to visualize and measure the percentage importance of different attributes with selected models. In brief, the questionnaire included 11 hypothetical scenarios. Respondents were asked to choose a preferred COVID-19 vaccination strategy for each scenario.

In addition, this study investigated the personality traits of respondents using the BFI-10 and classified respondents according to personality traits by LPA. We used a conditional logit model (CLOGIT) to assess and investigate the heterogeneity of respondents’ preferences for COVID-19 vaccines across personality traits.

### 2.2. Discrete Choice Experiment (DCE)

#### 2.2.1. DCE Overview

DCEs, also known as choice-based conjoint analysis (CBC), has become a commonly used instrument in health economics and patient preference analysis, addressing a wide range of policy questions [25]. The theoretical model for a DCE is based on the random utility model [26]. A DCE is a decomposition method, in that the implicit values of an attribute of intervention are derived from some overall score for a profile consisting (conjointly) of two or more attributes [27]. According to the consensus issued by the International Society for Pharmacoeconomics and Outcomes Research (ISPOR) on conjoint analysis, the steps of discrete choice experiments can be roughly divided into the following steps: research question, attributes, and levels, choice sets design (scenarios design), data collection and analyses, and results and conclusions. In this study, the design and implementation of the discrete choice experiment were conducted based on conjoint analysis consensus [27].

#### 2.2.2. Selection of Attributes and Levels

The objective of a DCE is to elicit preferences or values over the range of attributes and levels that define profiles in the DCE’s tasks. In the DCE, respondents were provided with several hypothetical scenarios (choice sets), each consisting of two or more alternatives. These alternatives are constructed by randomization with different attributes and levels. In each scenario, respondents choose that which, in their opinion, and in accordance with the random utility maximization framework, yields the highest utility; this, therefore, reflects their latent preferences as captured by the utility function [28]. In the field of health research, 4–6 attributes are the number of attributes that appear most frequently in the research [29], and 8–16 joint analysis tasks is the best number of tasks that respondents can perform [27].

We used the DCE to further explore respondents’ preferences for vaccination. The validity of the DCE questionnaire depends on reasonable attributes and levels. Vaccine attributes and their levels were identified and retrieved through the relevant literature and several vaccines on the market, and the attributes were then ranked, categorized, and refined by 13 experts in the fields of public health, vaccination, epidemiology, and psychology [30,31,32,33,34,35,36,37,38,39]. Finally, six attributes and their respective levels associated with COVID-19 vaccine preference were identified (Table 1).

#### 2.2.3. DCE Instrument Design

We used the fractional factorial design method to identify the optimal number of vaccination scenarios and designed this in Lighthouse Studio version 9.12.0 (Sawtooth Software, Orlando, FL, USA). In actual operation, if the full factorial design is adopted, there are six attributes in this study, with two to six levels for each attribute. Organizing levels under different attributes will result in 3600 combinations. (i.e., 3^2^ × 4^2^ × 5^2^ = 3600). In reality, it is unrealistic for respondents to answer 3600 combinations; thus, the fractional factorial method was essential in designing the DCE instrument. This method is based on the following two principles [26,40,41]: (1) orthogonality, which, in terms of the DCE, means that each attribute level should have little to no correlation with other attribute levels; and (2) balance, which means that each attribute should appear an equal number of times.

According to the rule of thumb as proposed by Johnson and Orme [42], the minimum sample size of a DCE study can be calculated with the formula of:

$$N\;>\;\frac{500c}{\left(t\;\times \;a\right)}$$

where *N* is the recommended minimum sample size, *t* is the number of selected tasks, *a* is the number of selections per task, and *c* is the maximum number of attribute levels. In this study, we calculated that *n* ≥ 64.1 (*t* = 13, A = 3, and C = 5). A total of 1200 valid samples were included in the study. The sample size is large, ensuring the accuracy and reliability of the calculation.

### 2.3. Data Collection

This study was a multi-stage sampling. Based on geographical division and population distribution, two to three provinces were selected from each of seven administrative regions in East China, South China, North China, Central China, Southwest China, Northeast China, and Northwest China by random number table method, totaling 18 provinces (autonomous regions, municipalities directly under the Central Government and special administrative regions).

Then we selected one to three cities from the selected provinces by the random number table method, but skipped this step if a municipality was directly under the central government. Then, based on the age distribution of population in China, quota sampling was carried out, such that the distribution of samples of all age groups conformed to the population characteristics. Two investigators were recruited for each city and, before the formal distribution of questionnaires, the investigators were given unified training. Each investigator was responsible for collecting 20–30 questionnaires. Inclusion criteria were for subjects 18 years of age and older without cognitive impairment (self-report). A quality test question was set in the DCE’s scenarios part of the questionnaire to ensure the quality of respondents’ answers. The questionnaire did not collect personally identifiable information. Survey data were collected from 15 July to 10 August 2021. The study was approved by the Jinan University Medical Ethics Committee JNUKY-2021-004).

### 2.4. Questionnaire Composition

#### 2.4.1. Demographic Characteristics and Vaccination Acceptance

In the first section of our survey, respondents were asked to provide socio-demographic information, including the respondents’ sex, age, education level, and annual income level. Further, respondents were asked to rate their acceptance of vaccination, with a scale of “totally unwilling, 0” to “totally willing, 10”. The detailed question was “How do you evaluate your willingness and acceptance to be vaccinated?” Respondents with an answer score of more than six were defined as having positive acceptance.

#### 2.4.2. The 10-Item Big Five Inventory (BFI-10)

The 10-item Big Five Inventory is a 10-item scale measuring the Big Five personality traits: Extraversion, Agreeableness, Conscientiousness, Neuroticism, and Openness [43]. The BFI-10 has five subscales with two bidirectional items for each of the big five personality factors. The items are valued on a five-point Likert scale ranging from “Strongly disagree” to “Strongly agree”. Each subscale was scored at 2–10 points. The values of Cronbach’s alpha ranged from 0.81–0.87, and Test-retest coefficients ranged from 0.52–0.87 in BFI-10 [44,45].

#### 2.4.3. DCE Scenarios

We used Lighthouse Studio (version 9.12.0, Sawtooth Software, Orlando, FL, USA) to generate 11 scenarios, each consisting of two hypothetical options and a “none” option (Table 2). The two hypothetical options were the options comprising attribute and level randomization, respectively. At the same time, to control the quality of the survey, we set up a logical error correction problem in the questionnaire; that is, we designed a combination with a fixed choice set to be superior to another combination in all attributes and levels. If the respondents chose the inferior scheme during the investigation, their answers were regarded as invalid.

The questionnaire was introduced with the following: When you are preparing for vaccination for COVID-19, you may be faced with the following scenarios about vaccines. Some of the scenarios may not meet the reality, but please choose one of the following three scenarios that you are most satisfied with according to the hypothetical situations and the existing information.

### 2.5. Statistical Analysis

#### 2.5.1. Latent Profile Analysis

We used LPA to explore the different personality portraits of the respondents according to their scores on the BFI-10 scale. The scores of sub-scales corresponding to Openness, Agreeableness, Neuroticism, Conscientiousness, and Extraversion in BFI-10 were used as explicit indicators. Using Mplus 8.0 software (Muthén & Muthén, Los Angeles, CA, USA), the number of profiles was increased sequentially starting from one profile, and model fitting for LPA was conducted. Multiple statistical indicators can be used to decide among models that differ in the number of classes. There are five different groups of statistical indices: Akaike Information Criteria (AIC), Bayesian Information Criteria (BIC), Adjusted Bayesian Information Criteria (aBIC), Entropy, and the Lombard-Rubin adjusted Likelihood Ratio Test (LMRT). Smaller values for AIC, BIC, and aBIC indicate better LPA fitting models. The value of Entropy is within 0–1, and closer to 1 indicates that the classification is more precise. The significant difference of LMRT (*p* < 0.05) indicated that the model with k class model was superior to the model with k-1 class [46]. Personality groups were determined according to LPA, and the naming of each group was based on the dimension with the most obvious characteristics in the group. When analyzing the most obvious features, we judged based on the differences of dimensions within and between groups. The variance analysis method was used to compare different groups to explore whether there were differences in demographic characteristics and COVID-19 vaccination willingness among respondents with different personality characteristics. CLOGIT and WTP were used to explore the preferences and willingness to pay for the attributes and levels of vaccines of respondents with different personality traits.

#### 2.5.2. Conditional Logit Model

Previous DCE studies applied the probit model, which assumes a normal distribution of the error term [47], or applied the logit model, which assumes a logistic distribution [48]. Due to its flexible applicability, the logit model is preferred in studies that use DCEs. The basis of the logit model is random utility theory. McFadden innovated upon the logit model and named this new model the “conditional logit” model. Conditional logit was shown by McFadden [49] to be consistent with random utility theory. He applied this model to choice behavior consistent with economic theory and derived a regression model which linked choices with the characteristics of alternatives available to decision makers [50].

Using random utility theory, the utility associated with an alternative or profile is assumed to be a function of observed characteristics (attribute levels) and unobserved characteristics of the alternative. This theoretic framework also assumes that each individual, when faced with a choice between two or more alternatives, will choose the alternative that maximizes their utility. The utility function is specified as an indirect utility function defined by the attribute levels in the alternative plus a random error term reflecting the researcher’s inability to perfectly measure utility:

$$U_{i}\;=\;(\beta ,X_{i})\;+\;\varepsilon_{i}$$

where *U* is a function defined by the attribute levels for alternative *i*, *ε_i_* is a random error term, *X*_i_ is a vector of attribute levels defining alternative *i*, and *β* is a vector of estimated coefficients. Each estimated coefficient is a preference weight and represents the relative contribution of the attribute level to the utility that respondents assign to an alternative. In conditional logit, *ε_i_* is assumed to follow an independently and identically distributed type 1 extreme-value distribution [49].

The assumption of the extreme-value distribution of *ε_i_* results in a logit model:

(1) $$Pr(choice\;=\;i)\;=\;\frac{e^{\nu (\beta ,x_{i})}}{\sum_{j}e^{\nu (\beta ,x_{j})}}$$

where *ν*(*β*, *x_i_*) is the observed portion of the function for alternative *i*, and *i* is one alternative among a set of *j* alternatives. Simply stated, the probability of choosing alternative *i* is a function of both the attribute levels of alternative *i* and the attribute levels of all other profiles presented in a choice task. In the case of the two-alternative, forced-choice DCE, there are two alternatives in each choice task, and so *j* ¼ 2. The probability of choosing one profile from the set of two alternatives is 1 minus the probability of choosing the other profile in that choice task. Therefore, neither alternative in the choice task has a choice probability of less than 0% or greater than 100%. In addition, this specification implies that the closer the probability of choosing an alternative in a two-alternative choice task is to 50%, the more sensitive the probability of choosing that alternative is to changes in the attribute levels that define the alternative [50].

In the DCE Data Analysis section, we used the conditional logit model (CLOGIT) to quantify preference heterogeneity in vaccine attributes and trade-off levels among respondents with different personality characteristics, which was analyzed in Lighthouse Studio (version 9.12.0, Sawtooth Software, Orlando, FL, USA). We virtually encoded attributes and levels to calculate preference heterogeneity across different classes, including β, *p*-value, ORs, 95%CI, where ORs and 95%CI were calculated by selecting a level in each attribute as the reference level. The CLOGIT provides statistical inferences about the preference weights of the respondents for each attribute and level included in the questionnaire. The sign of that coefficient (positive or negative) indicates the preferred direction of respondents for a given attribute level.

#### 2.5.3. Willingness to Pay

Willingness to pay (WTP) is an efficient metric for measuring how much an individual is willing to sacrifice (i.e., economic sacrifices) to choose one diagnosis attribute level over another (i.e., the reference attribute level). WTP can be derived from a DCE. WTP analyses via DCE have been applied to a wide range of interventions in different markets, including vaccine research [51,52,53,54,55,56,57]. A body of literature has evaluated individuals’ WTP for hypothetical vaccines, including, for example, dengue [51], HIV [52], and influenza [53]. Recently, several authors have evaluated individuals’ WTP for, and acceptance of, a COVID-19 vaccine in Europe [54], Asia [55], Africa [56], and America [57]. We analyzed the WTP of Chinese respondents with different personality properties to identify homogeneity and heterogeneity in participants’ preferences.

## 3. Results

### 3.1. General Information and Latent Profile Analysis

A total of 1465 respondents completed the questionnaire. After questionnaires with missing data, quality problems, and age noncompliance were excluded, a total of 1200 responses were included in the study and the effective rate was 81.9%. Of the 1200 respondents, 1200 (100%) were aged 18–85 years, 701 (58.4%) were female, 495 (41.3%) were male, and 4 (0.3%) were of other genders, 243 (20.3%) did not have a high school diploma, 957 (79.3%) had a high school or above diploma, 491 (40.9%) were unmarried, and 640 (53.3%) were married.

We identified the model containing the optimal number of profiles using the criteria outlined by Nylund et al. [58]. According to the guidelines of Nylund et al., we started with a two-profile model and added profiles through an iterative process, noting the fit statistics for each model. The indices for the LPA and profile structure are shown in Table 3. The *p*-value for LMRT was not significant for 2, 4, and 5 profiles, and the LMRT was significant for 3 profiles (*p* < 0.05), so the model is the best when the number of profiles is 3 (Table 3).

**Figure 1:**
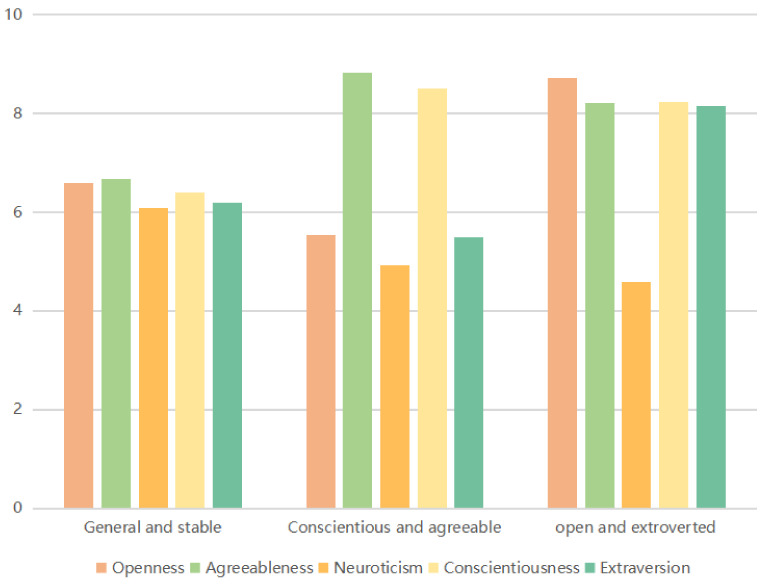
Descriptive statistics for the three-profile model.

The three-profile model, according to the measurement results of the 10-item Big Five Inventory, divides all respondents into three different types of personality portraits, as shown in Figure 1. In total, 79.67% (956/1200) of respondents are typified by having the highest scores of Neuroticism (6.092 ± 1.241) and medium scores of Agreeableness (6.686 ± 1.141), Openness (6.584 ± 1.346), Conscientiousness (6.397 ± 1.316), and Extraversion (6.198 ± 1.398). With little difference in scores between the five personality traits in profile 1, of which this group has the highest proportion, this can be described as the General and Stable type. Then, 9.5% (114/1200) of respondents are typified by having the highest scores of Agreeableness (8.833 ± 0.808) and Conscientiousness (8.5 ± 1.091), medium scores of Openness (5.535 ± 1.506) and Extraversion (5.491 ± 1.746), and the lowest of Neuroticism (4.921 ± 1.63). Compared with the other two groups, Agreeableness and Conscientiousness scored highest. This group can thus be characterized as the Conscientious and agreeable type. Lastly, 10.83% (130/1200) of respondents had the highest score of Openness (8.715 ± 1.163), moderate scores of Conscientiousness (8.238 ± 1.504), Agreeableness (8.215 ± 1.148), and Extraversion (8.154 ± 1.32), and the lowest score of Neuroticism (4.538 ± 1.516). Openness and Extraversion scored highest compared to the other groups. This group can be characterized as the Open and Extroverted type.

In the three kinds of personality portraits, there were no significant differences in gender, age, education level, or annual income level (*p* > 0.05), except for the score of willingness to receive a COVID-19 vaccine (*p* < 0.05), as shown in Table 4. In terms of willingness to receive a COVID-19 vaccine, all three categories of respondents had better willingness to vaccinate with scores higher than 5 (9.03 scores). The Open and Extroverted type has the highest willingness to vaccinate (9.446 scores), followed by the Conscientious and Agreeable type (9.175 scores), and the lowest score was the General and Stable type (8.959 scores).

### 3.2. DCE Results

**Figure 2:**
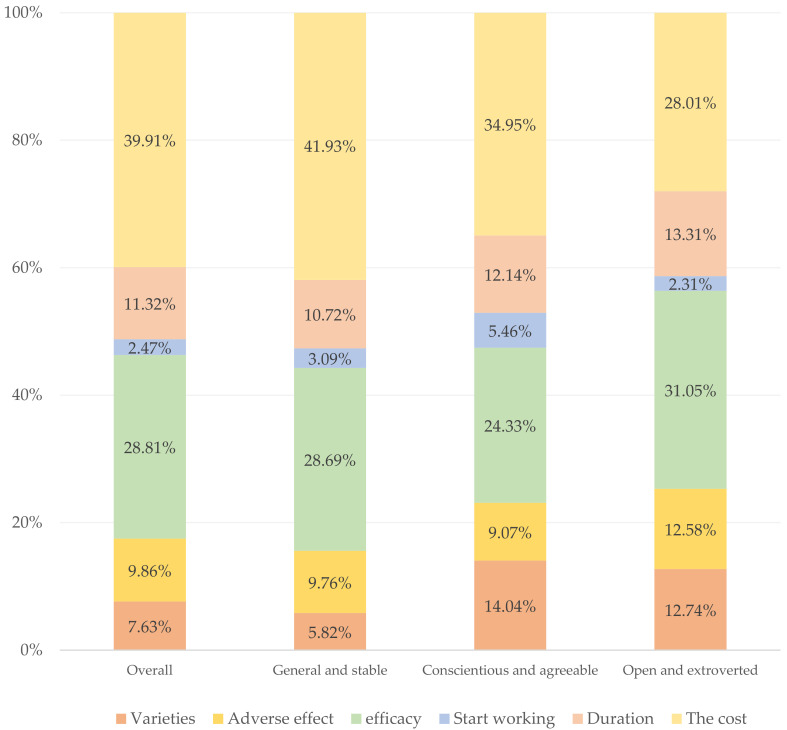
Percentage importance of attributes in latent profile analysis.

Regarding the percentage importance of vaccine attributes, the General and Stable type and the Conscientious and Agreeable type respondents considered the most important attribute to be vaccine cost (General and Stable type: 41.93%; Conscientious and Agreeable type: 34.95%), while the Open and Extroverted type respondents considered vaccine efficiency to be the most important (31.05%). For the second most important attribute, other types of respondents considered vaccine efficiency (General and Stable type: 28.69%; Conscientious and Agreeable type: 24.33%), except for Open and Extroverted respondents who considered vaccine cost to be important (28.01%), as shown in Figure 2.

According to the ORs of overall respondents and three types of respondents with different personality characteristics (Table 5), all categories of respondents preferred inactivated vaccines and least preferred adenovirus vector vaccines. Regarding vaccine side effect attributes, the Open and Extroverted respondents preferred “mild” adverse effects the most (OR 1.108, 95% CI 0.977–1.256), General and Stable type and Conscientious and Agreeable type respondents preferred “very mild” adverse effects, and all categories of respondents disliked “moderate” adverse effects. In the vaccine start time attribute, the “20 days” start time for overall respondents and the “15 days” and “20 days” start times for General and Stable respondents were statistically preferred (*p* < 0.05). The General and Stable respondents preferred “15 days” start time to “20 days” start time (15 days: OR1.073, 95% CI 1.012–1.138; 20 days: OR 0.948, 95% CI 0.894–1.006).

### 3.3. Overall WTP

Results of the WTP estimation supported the comparisons of the respondents’ preferences from a monetary perspective. By analyzing the willingness to pay of the three groups of respondents with different personality traits, we found that all types of interviewees were willing to pay the highest cost for the inactivated vaccine, and the Open and Extroverted respondents were willing to pay the highest cost (75.10 USD). Overall, the General and Stable type and Open and Extroverted type respondents were willing to pay more for “very mild” vaccine side effects, with the General and Stable respondents willing to pay the most (55.96 USD). The Open and Extroverted respondents were willing to pay more for “mild” vaccine side effects (63.57 USD). For vaccine efficacy, all categories of respondents were willing to pay the highest amount for 95% of vaccine efficacy, with the Open and Extroverted respondents having the highest willingness to pay (162.34 USD) among the three groups of respondents with different personality traits. The attribute for the time for the vaccine start working was not analyzed in depth because most of the data in the conditional logit model were not statistically significant (Table 6). For vaccine duration, all categories of respondents had the highest willingness to pay for “20 months”, with the Open and Extroverted respondents having the highest willingness to pay (80.79 USD). The analysis of the WTP of the three categories of respondents with different personality traits revealed that the Open and Extroverted respondents were more willing to pay more for the most preferred level of the COVID-19 vaccine.

## 4. Discussion

### 4.1. Principal Results

This study provides concrete evidence for the relationship between personality traits and the Chinese public’s willingness and preference for vaccination against COVID-19. In this study, we investigated the effects of personality characteristics among the Chinese general public on their preferences for COVID-19 vaccination. Based on the BFI-10, respondents were categorized into three personality groups using LPA. We compared the demographic characteristics (i.e., gender, age, highest education level, and annual income) of the three groups of respondents and found no statistical differences. Therefore, this study eliminates the influence of demographic and sociological characteristics on the findings to some extent and was more conducive to focusing on the influence of personality characteristics on the Chinese public’s preferences for COVID-19 vaccination.

In terms of willingness to vaccinate against COVID-19, the Chinese public is generally willing to vaccinate. In a concise systematic review of global COVID-19 vaccine hesitation and vaccine acceptance rates, surveys on COVID-19 vaccine acceptance rate were found in 33 different countries. Among adults representing the public, Ecuador (97.0%), Malaysia (94.3%), Indonesia (93.3%), and China (91.3%) had the highest acceptance rates of COVID-19 vaccines [59]. Although the Chinese public is generally willing to vaccinate, there are still differences in willingness to vaccinate against COVID-19 among Chinese public groups with different personality traits.

The Open and Extroverted type had the highest willingness to accept, followed by the Conscientious and Agreeable type, and the lowest was seen in the General and Stable type. For the Open and Extroverted type respondents, more Open individuals tend to seek out new, nontraditional ideas and experiences and are flexible, curious, and creative [58,60,61]. Thus, during the COVID-19 pandemic, more Open individuals may have less difficulty adapting to a new situation and find more nontraditional ways to cope with it. The use of COVID-19 vaccines, as one of the means of preventing infection with COVID-19, is an approach that requires gradual public acceptance of recognition, and more open-minded respondents are more likely to accept and adapt to new measures. Extraversion reflects positive emotions, optimism, etc., and the degree to which a person needs attention and socialization [58]. Extroverted individuals are more likely to have intimate relationships and social contact [62]. Ensuring Extroverts’ compliance with guidelines to slow the spread of COVID-19 is challenging, especially as it seems difficult to maintain social distancing [63]. The needs to maintain social distance, reduce social activities, and isolate at home during an outbreak, among other preventive measures, are not short-term requirements but are long-term in nature. This can be difficult for Extroverted people. However, the establishment of herd immunity by vaccination against COVID-19 can somewhat reduce the restrictions on home isolation and maintaining social distance.

For the Conscientious and Agreeable type respondents, Conscientious individuals tend to be responsible, dutiful, and self-disciplined [58,64]. Studies have shown that high Conscientiousness is associated with taking more COVID-19 precautions [65], the ability to fight viruses in strict compliance with government rules and recommendations [23], and the adoption of beneficial health behaviors [66]. Agreeable individuals tend to be kind, cooperative, and respectful [58]. During the COVID-19 pandemic, more Agreeable individuals thus comply more strictly with socially desirable safety rules and strive to support and protect others to maintain positive relationships with them [23]. A study investigated the relationship between Big Five personality traits and individual perception of vaccination in a sample of 3276 American citizens and found that individuals with a higher sense of responsibility and affinity also showed a more positive attitude towards vaccination [24].

For the General and Stable type of respondents, Neuroticism levels were higher than for the other two types of respondents. Individuals with high Neuroticism were less emotionally stable and more prone to anxiety, anger, depression, impulsivity, and overwhelming stress [58,67]. One study has found that higher Neuroticism in the United States was associated with more COVID-19 concerns and fewer preventive measures [65]. Lower Neuroticism and higher Extroversion, Agreeableness, Openness, and Conscientiousness were associated with stricter adherence to COVID-19 guidelines [68]. The above reasons may explain the relatively low willingness to vaccinate among the General and Stable respondents compared to the other two types.

Regarding the percentage importance of vaccine attributes, the General and Stable and the Conscientious and Agreeable types of respondents considered vaccine cost to be the most important and vaccine efficacy to be the second most important attributes. This was consistent with the percentage importance of attributes perceived by all respondents. At the same time, it is also consistent with the general preference of the Chinese public in a study on the hesitation of the Chinese and American public to vaccinate against COVID-19. However, the Open and Extroverted type was the opposite of the other two types, with this type considering vaccine efficacy to be the most important and vaccine cost coming in second place. We speculate that this was because of the higher Extroversion scores in this type. The need for contact and socialization with other people is higher in Extroverted individuals [62], so the effectiveness of the COVID-19 vaccination in protecting respondents from normal social contact was of greater concern in this type.

The results of the CLOGIT showed that for vaccine varieties, the three groups of respondents preferred inactivated vaccines, which may be related to the varieties of vaccines available to the respondents. At present, five production enterprises in China have received approval for the conditional marketing or emergency use of their COVID-19 vaccines, including three inactivated vaccines, one adenovirus vector vaccine, and one recombinant COVID-19 vaccine (CHO cells) [69]. It has been shown that the incidence of adverse reactions as a side effect of the COVID-19 vaccines is roughly as follows: adenovirus vector vaccine > mRNA vaccine > recombinant protein vaccine > inactivated vaccine [70]. This may explain why the three groups of respondents preferred inactivated vaccines the most and adenovirus vaccines the least. For vaccine side effects, the General and Stable type and the Conscientious and Agreeable type respondents preferred “very mild” adverse effects, which was consistent with our hypothesis. However, the Open and Extroverted respondents preferred “mild” adverse effects, which was inconsistent with our conjecture. We suspect that the “mild” adverse effects of the vaccine may be seen as reflecting the fact that the vaccine was working in the body. “Mild” side effects (e.g., fatigue, colds, etc. [7]) were considered acceptable by this type of respondent. Moreover, among the three types of respondents, the Open and Extroverted type had the lowest Neuroticism score, had the fewest preferences influenced by Neuroticism, and thus did not attach as much importance to vaccine side effects as the other two types of respondents. However, the above explanation is only speculative and requires further in-depth study.

By analyzing the WTP of the three types of respondents for the COVID-19 vaccine, we found that the WTP of the three types of respondents was more consistent with the results of the CLOGIT analysis and that the three types of respondents were willing to pay higher amounts for their most-preferred attributes and levels. More importantly, our study found that for the most-preferred vaccine levels, the Open and Extroverted type respondents had higher WTP values than the General and Stable type and the Conscientious and Agreeable type. A German study of WTP in health insurance identified a link between personality factors (i.e., openness to experience) and WTP and found that openness to experience appeared to be associated with an increase in WTP [71]. The fact that the Open and Extroverted type had the highest WTP among the three types of respondents may also explain why the Open and Extroverted interviewees did not consider the vaccine cost to be the most important attribute in the percentage importance of the vaccine attribute, unlike the other two types. Based on the above study, the three types of respondents with different personality traits showed heterogeneity in preferences for COVID-19 vaccination willingness, percentage importance of vaccine attributes, vaccine attributes and levels, and WTP for vaccine attributes and levels.

### 4.2. Suggestion

Personality precedes any particular attitude or behavior in causality and is an important determinant of various attitudes and behaviors. When accurate information about vaccination is provided, people with certain personality characteristics are more likely to know or accept this information, and further change their attitude towards vaccination [19].

The government and relevant authorities may consider starting with personality traits when proposing initiatives to enhance public vaccination against COVID-19. This could involve a focus on the public’s personality traits and the different ideas and behaviors of COVID-19 vaccination preference due to different personalities [72], and taking more personalized and targeted measures according to the public’s personality traits. In particular, when disseminating health information about the COVID-19 vaccines, different levels of emphasis should be placed on the vaccines’ attributes and levels preferred by the public with different personality traits. A study on psychological characteristics related to hesitation and drug resistance of COVID-19 vaccine in Ireland and the United Kingdom suggested that people who are hesitant or drug-resistant towards a vaccine are usually serious, emotionally unstable, weak in analytical ability, and not easy-going, and the public health information for these people should be clear, direct, repetitive and positive [73,74].

For the General and Stable and the Conscientious and Agreeable members of the public, vaccine cost is the most preferred attribute for these two types. Currently, it is beneficial that many countries have financed their immunization programs, making COVID-19 vaccines free of charge for the public [75,76]. Governments can make use of the media as an effective means of publicity, and adopt diversified and vivid methods to disseminate vaccine-related knowledge. The government should make the public fully aware of the consequences of COVID-19 infection and the benefits of vaccination, to establish mass immunization. Especially for the General and Stable and slightly low-willing public, it is necessary to establish the public’s correct understanding of the COVID-19 vaccines and improve the public’s willingness to accept COVID-19 vaccines. The government should not only simply inform the public that vaccines are safe through the media, but also should thoroughly popularize the similarities and differences of different kinds of vaccines in terms of technology and clinical effects to break wrong public opinions. The government is mainly responsible for health education such as COVID-19 vaccination for the public. Meanwhile, health care personnel, scientists, and social stakeholders should also exert their efforts to pass on the health knowledge of the COVID-19 vaccines and create a good atmosphere for vaccination for themselves and society. For the Open and Extroverted members of the public, in addition to the recommendations and measures applicable to the above, the government and relevant authorities should publish timely information on the progress of COVID-19 vaccine development, its effectiveness for the prevention of SARS-CoV-2 and variants, possible adverse effects, and the duration of vaccine effectiveness. In addition, information on vaccine safety surveillance should be published regularly and timely health education should be provided through authoritative channels.

### 4.3. Strengths and Limitations

In this study, we delved into the personality perspective of respondents, classified all respondents according to the Big Five personality traits using latent profile analysis, and explored the heterogeneity of preferences for COVID-19 vaccine among respondents with different personality traits using a discrete choice experiment. There were no significant differences among all groups of respondents in terms of gender, age, highest education, and annual income. This could well exclude the effect of demographic characteristics on the preference of respondents with different personality traits in terms of vaccine attributes and levels. In addition, an extensive literature search was conducted to determine the attributes and levels of the DCE, and a group of experts in the field of public health and vaccination was invited to conduct interviews to further revise and refine the attributes and levels.

The present study has some limitations. First, China has a large population and our sample data cannot fully represent the actual situation of the whole of China, and there is a problem of sample selection bias, which may affect the results of our analysis. Second, although the attributes and levels of the DCE were determined based on a literature survey and 13 experts in the fields of public health, vaccination, epidemiology, and psychology, the assumptions and scenarios presented in the questionnaire may not exactly match the reality of the actual situation. In addition, our research only includes the first six attributes that experts think are the most important, ignoring other factors that may affect vaccination. Third, most Chinese people have been vaccinated with the vaccines independently developed by China. In this study, we pay attention to the Chinese public’s preference for different types of vaccines, while ignoring the influence of vaccines produced in different countries on Chinese public choice. Fourthly, this study investigated respondents’ self-reported vaccination preferences rather than the objective reality, which may have affected the objectivity of this study.

## 5. Conclusions

Respondents with different personality traits showed preference heterogeneity in COVID-19 vaccination intentions, percentage importance of vaccine attributes, adverse vaccine reactions, and WTP of vaccine attributes and levels. In terms of willingness to be vaccinated against COVID-19, the Open and Extroverted members of the public had the highest willingness to vaccinate. In terms of percentage importance of attributes, the General and Stable and Conscientious and Agreeable type respondents thought that vaccine cost was the most important attribute, while the Open and Extroverted type thought that vaccine effectiveness was the most important attribute. In the CLOGIT model, there were differences in the preferences of the three types of respondents for the level of vaccine side effects. The General and Stable type and Conscientious and Agreeable type respondents preferred the “mildest” side effects, while the Open and Extroverted type preferred “mild” side effects. In our WTP analysis, we found that compared with other types, the Open and Extroverted respondents had a higher willingness to pay for their favorite vaccine level. It is suggested that the government and related departments should pay more attention to the vaccine attributes and levels of concern to the public with different personality traits, and develop individualized and targeted vaccination strategies for groups of the public with different personality traits, especially in regards to the dissemination of health information about COVID-19 vaccines, with different emphasis for groups of the public with different personality traits.

## Figures and Tables

**Table 1:** Vaccination of attributes and their respective levels in this discrete choice experiment.

Attributes	Levels of Attributes	Explain
Vaccine varieties	mRNA vaccine	Several common vaccines are produced by countries at present.
	Adenovirus vector vaccine	
	Inactivated vaccine	
Adverse effect	Very mild	The extent of possible side effects of vaccination with COVID-19.
	mild	
	Moderate	
Efficacy	55%	Effectiveness of vaccination against infection with COVID-19
	65%	
	75%	
	85%	
	95%	
Time for the vaccine to start working	5 days	Time from the time the COVID-19 vaccine is administered until the time the vaccine begins to work in the body.
	10 days	
	15 days	
	20 days	
The duration of vaccine works	5 months	Duration of time that a COVID-19 vaccine takes effect in the body until it ceases to be effective.
	10 months	
	15 months	
	20 months	
The cost of vaccination	$0	The total cost of vaccinating COVID-19
	$50	
	$100	
	$150	
	$200	

**Table 2:** An example scenario of the choice-based conjoint in the questionnaire; scenario #1/11.

Attributes	Vaccine A	Vaccine B	Neither
Vaccine varieties	Inactivated vaccine	mRNA vaccine	Neither
Adverse effect	mild	Very mild	
Efficacy	95%	55%	
Time for the vaccine to start working	20 days	10 days	
The duration of vaccine works	5 months	20 months	
The cost of vaccination	$0	$0	

**Table 3:** Latent Profile Model Information Criteria, Likelihood Ratio Test, and Entropy (N = 1200).

Number of Profiles	AIC	BIC	ABIC	Entropy	*p*-Value of LMRT
1	21,759.116	21,810.017	21,778.253		
2	21,535.778	21,617.22	21,566.397	0.677	0.6492
3	21,439.248	21,551.23	21,481.349	0.722	0.0215
4	21,405.553	21,548.076	21,459.137	0.708	0.1956
5	21,359.904	21,532.967	21,424.97	0.719	0.1031

**Table 4:** Demographic characteristics of different personality portraits.

Demographic Items	General and Stable Type (*n* = 956)	Conscientious and Agreeable Type (*n* = 114)	Open and Extroverted Type (*n* = 130)	F Value	*p*-Value
Gender (%)				2.149	0.117
Male	408 (56.1%)	37 (32.5)	50 (38.5%)		
Female	544 (33.3%)	77 (67.5)	80 (61.5%)		
Other	4 (7.9%)	0 (0%)	0 (0%)		
Age interval in years (%)				2.164	0.115
18–40	592 (61.9%)	54 (47.4)	87 (66.9%)		
41–60	225 (23.5%)	48 (42.1)	29 (22.3%)		
Above 60	139 (14.5%)	12 (10.5)	14 (10.8%)		
Highest educational level (%)				2.376	0.093
Pre-primary education or primary school education	77 (8.1%)	12 (10.5%)	4 (3.1%)		
Middle school education	127 (13.3%)	11 (9.6%)	12 (9.2%)		
High school education	159 (16.6%)	11 (9.6%)	25 (19.2%)		
Vocational school education	164 (17.2%)	33 (28.9%)	16 (12.3%)		
Bachelor’s degree	360 (37.7%)	41 (36%)	66 (50.8%)		
Master’s degree	56 (5.9%)	4 (3.5%)	6 (4.6%)		
Ph.D. degree	13 (1.4%)	2 (1.8%)	1 (0.8%)		
Annual salary level (%)				1.447	0.236
Under USD 10,000	619 (64.7%)	64 (56.1%)	86 (66.2%)		
USD 10,001–30,000	261 (27.3%)	38 (33.3%)	34 (26.2%)		
USD 30,001–50,000	52 (5.4%)	9 (7.9%)	8 (6.2%)		
Above USD 50,000	24 (2.5%)	3 (2.6%)	2 (1.5%)		
Acceptance of vaccination (totally unwilling, 0–totally willing, 10)				4.669	0.01
Average	8.96	9.18	9.45		

**Table 5:** General results of the conditional logit model. Comparison of overall and three categories of respondents with different personality traits in attributes levels utility and odds ratios.

Attributes and Levels	Overall, *n* = 1200 (100%)		General and Stable Type,		Conscientious and Agreeable Type, *n* = 114 (9.5%)		Open and Extroverted Type, *n* = 135 (10.7%)	
			*n* = 956 (79.7%)					
	Coefficient	Odds Ratio (95%CI)	Coefficient	Odds Ratio (95%CI)	Coefficient	Odds Ratio (95%CI)	Coefficient	Odds Ratio (95%CI)
Varieties								
mRNA vaccines	−0.081 ***	REF	−0.064 **	REF	−0.183 *	REF	−0.126	REF
Adenovirus vector vaccines	−0.117 ***	0.964 (0.925–1.006)	−0.085 ***	0.979 (0.934–1.026)	−0.255 ***	0.930 (0.810–1.069)	−0.236 ***	0.896 (0.788–1.020)
Inactivated vaccines	0.198 ***	1.32 (1.267–1.376)	0.149 ***	1.237 (1.181–1.296)	0.437 ***	1.859 (1.624–2.128)	0.362 ***	1.629 (1.436–1.848)
Adverse effect								
very mild	0.153 ***	REF	0.156 ***	REF	0.165 *	REF	0.129 *	REF
mild	0.099 ***	0.947 (0.909–0.988)	0.080 **	0.926 (0.884–0.971)	0.118	0.954 (0.833–1.093)	0.231 ***	1.108 (0.977–1.256)
moderate	−0.253 ***	0.666 (0.639–0.695)	−0.236 ***	0.676 (0.644–0.709)	−0.283 ***	0.639 (0.554–0.737)	−0.359 ***	0.614 (0.538–0.700)
Efficacy								
55%	−0.601 ***	REF	−0.574 ***	REF	−0.726 ***	REF	−0.700 ***	REF
65%	−0.282 ***	1.375 (1.292–1.464)	−0.289 ***	1.330 (1.239–1.428)	−0.155	1.770 (1.444–2.169)	−0.387 ***	1.367 (1.129–1.656)
75%	0.008	1.839 (1.731–1.953)	−0.028	1.726 (1.612–1.848)	0.165	2.438 (2.006–2.962)	0.150	2.340 (1.947–2.811)
85%	0.289 ***	2.435 (2.295–2.583)	0.312 ***	2.426 (2.270–2.592)	0.244 *	2.638 (2.166–3.213)	0.180	2.410 (2.010–2.888)
95%	0.585 ***	3.273 (3.086–3.473)	0.579 ***	3.166 (2.963–3.383)	0.473 ***	3.318 (2.725–4.040)	0.756 ***	4.289 (3.578–5.140)
Start working								
5 days	−0.017	REF	−0.010	REF	−0.160	REF	0.049	REF
10 days	0.021	1.039 (0.987–1.095)	0.013	1.024 (0.966–1.085)	0.109	1.309 (1.104–1.552)	0.002	0.954 (0.815–1.116)
15 days	0.049	1.069 (1.015–1.125)	0.060 *	1.073 (1.012–1.138)	0.020	1.197 (1.007–1.424)	0.009	0.961 (0.822–1.124)
20 days	−0.053 *	0.965 (0.916–1.017)	−0.064 *	0.948 (0.894–1.006)	0.030	1.210 (1.017–1.438)	−0.059	0.897 (0.767–1.049)
Duration								
5 months	−0.282 ***	REF	−0.274 ***	REF	−0.332 ***	REF	−0.317 ***	REF
10 months	−0.005	1.319 (1.252–1.389)	0.002	1.318 (1.244–1.398)	−0.024	1.361 (1.145–1.618)	−0.012	1.356 (1.160–1.585)
15 months	0.104 ***	1.471 (1.397–1.549)	0.115 ***	1.476 (1.393–1.564)	0.089	1.523 (1.283–1.807)	0.021	1.402 (1.199–1.640)
20 months	0.184 ***	1.593 (1.514–1.677)	0.157 ***	1.538 (1.452–1.630)	0.267 **	1.819 (1.538–2.152)	0.308 ***	1.867 (1.600–2.178)
The cost								
$0	0.924 ***	REF	0.948 ***	REF	0.974 ***	REF	0.751 ***	REF
$50	0.217 ***	0.493 (0.465–0.523)	0.232 ***	0.489 (0.458–0.522)	0.160	0.443 (0.365–0.538)	0.157	0.522 (0.461–0.661)
$100	−0.128 ***	0.349 (0.329–0.371)	−0.151 ***	0.333 (0.311–0.357)	−0.126	0.333 (0.272–0.407)	0.032	0.487 (0.406–0.585)
$150	−0.294 ***	0.296 (0.278–0.315)	−0.292 ***	0.290 (0.270–0.311)	−0.258 *	0.292 (0.238–0.358)	−0.378 ***	0.323 (0.267–0.391)
$200	−0.719 ***	0.193 (0.181–0.207)	−0.737 ***	0.186 (0.172–0.200)	−0.749 ***	0.179 (0.143–0.224)	−0.562 ***	0.269 (0.221–0.328)

REF: Reference level; * *p* < 0.05 ** *p* < 0.01 *** *p* < 0.001.

**Table 6:** WTP ^a^ of respondents with different personality traits ^b^.

Attributes and Levels	Overall (*n* = 1200) (USD)	General and Stable Type (*n* = 951) (USD)	Conscientious and Agreeable Type (*n* = 114) (USD)	Open and Extroverted Type (*n* = 135) (USD)
Varieties				
Adenovirus vector vaccines	REF	REF	REF	REF
mRNA vaccines	1.84	0.08	8.03	3.96
Inactivated vaccines	26.25	34.45	59.18	75.10
Adverse effect				
moderate	REF	REF	REF	REF
very mild	30.55	55.96	50.00	44.80
mild	20.00	38.04	48.54	63.57
Efficacy				
55%	REF	REF	REF	REF
65%	9.35	17.55	25.15	21.09
75%	16.58	31.51	40.75	84.28
85%	66.38	73.83	62.61	88.28
95%	222.36	115.53	94.86	162.34
Start working				
5 days	REF	REF	REF	REF
10 days	−0.38	−3.87	30.18	3.81
15 days	0.20	−0.48	23.98	−1.46
20 days	−3.38	−6.50	18.75	0.59
Duration				
5 months	REF	REF	REF	REF
10 months	19.22	37.23	7.68	37.01
15 months	33.28	68.80	31.98	50.29
20 months	62.28	75.39	61.72	80.79

^a^ WTP: willingness to pay; ^b^ Negative currency values refer to the amount that respondents were willing to pay for another level.

## Data Availability

Data is contained within the article.
